# Facilitators and barriers to smoking cessation support among professionals in social and community service settings: a systematic review and thematic synthesis

**DOI:** 10.1093/her/cyaf030

**Published:** 2025-08-12

**Authors:** Judith E M Visser, Fatima A Nur, Andrea D Rozema, Anton E Kunst, Mirte A G Kuipers

**Affiliations:** Department of Public and Occupational Health, Amsterdam Public Health Research Institute, Amsterdam UMC, University of Amsterdam, Van der Boechorststraat 7, 1081 BT Amsterdam, The Netherlands; Department of Public and Occupational Health, Amsterdam Public Health Research Institute, Amsterdam UMC, University of Amsterdam, Van der Boechorststraat 7, 1081 BT Amsterdam, The Netherlands; Tranzo Scientific Center for Care and Wellbeing, Tilburg School of Social and Behavioral Sciences, Tilburg University, Professor Cobbenhagenlaan 125, 5037 DB Tilburg, The Netherlands; Department of Public and Occupational Health, Amsterdam Public Health Research Institute, Amsterdam UMC, University of Amsterdam, Van der Boechorststraat 7, 1081 BT Amsterdam, The Netherlands; Department of Public and Occupational Health, Amsterdam Public Health Research Institute, Amsterdam UMC, University of Amsterdam, Van der Boechorststraat 7, 1081 BT Amsterdam, The Netherlands

## Abstract

Social and community service settings are a promising environment to support individuals with lower socioeconomic positions in quitting smoking. However, there remains a notable lack of support from their professionals in these settings. This study provides an overview of facilitators and barriers to smoking cessation support among these professionals. A systematic review was conducted up to April 2024 using five databases. Data were analysed using thematic synthesis, with themes categorized according to the Social Ecological Model. Eleven studies were included. We found twelve factors that could facilitate professionals in providing support. These factors related to the intrapersonal (i.e. knowledge/skills, self-efficacy, and belief), interpersonal (i.e. trustworthy connection with clients, readiness of clients, and clients’ supportive social environment), organizational (i.e. expertise improvement in smoking cessation, availability of resources, and organizational support), and societal level (i.e. availability of appropriate cessation programmes, supportive healthcare financing, and public awareness). We found that these factors often were not present, which hindered professionals from providing support. Professionals working in social and community service settings could reach many people who smoke. However, there are numerous obstacles to overcome before their full potential can be realized. To harness this potential, organizational changes are necessary, with governments playing a supportive role.

## Introduction

Worldwide, tobacco causes over 8 million deaths every year, including approximately 1.3 million non-smokers exposed to secondhand smoke [1]. Tobacco contributes to approximately half of the inequalities by socioeconomic position (SEP; measured by social class, income, or education) in death rates and life expectancy [2]. Particularly in high-income countries, smoking rates have declined more rapidly among individuals from higher socioeconomic backgrounds, leading to increased disparities in smoking-related health problems [1, 3–6]. For example, in Europe, higher-educated individuals were approximately half more likely to quit smoking compared to those with lower education levels between 2002 and 2012 [6]. In 2020, the average smoking rate among higher educated individuals was 13.4% compared to 19.4% among those with lower education levels [7].

One possible reason for the difference in quit success rates is that people with low SEP appear to have limited access to smoking cessation programmes that are generally the most effective [8]. Several barriers have been identified, including the unavailability of local support, financial difficulties, and insufficient access to intensive and flexible support [8, 9]. To improve access to effective smoking cessation support, additional strategies should take these barriers into account.

One strategy could be to intervene in environments with direct access to people with a low SEP, for example in Social and Community Service (SCS) settings [10]. Worldwide, organizations in these settings can be both governmental and non-governmental and provide social welfare services to improve quality of life and enable full participation in society, primarily for disadvantaged groups such as low SEP individuals [11–13]. Depending on the region, these organizations may offer services and facilities related to work, participation, self-reliance, housing, food aid, and support for children and families, as well as providing financial and material assistance. In Australia and the United States, these settings have been shown to be a novel community-based environment for reaching people with a low SEP and supporting them to quit smoking [13, 14]. Professionals in these settings have existing relationships with people from low SEP groups, with over half of their clients being current smoker (56%). They are committed, provide holistic care, and often have regular and long-standing contact with their clients [11, 15, 16].

Professionals working in SCS settings could undertake several activities to improve access to effective smoking cessation support services [10, 17, 18]. These activities include addressing tobacco use, advising or motivating clients to quit, and refer clients to smoking cessation support, such as the general practitioner or a specialized smoking care provider. Additionally, professionals may organize or facilitate smoking cessation programmes or personally counsel clients during their quit attempt.

In one study, over half of the smoking clients in SCSs expressed a desire for support from staff members to quit smoking [19]. Another study found that integrating smoking cessation care in these settings could have a positive impact on client smoking, including their quitting intentions and behaviour [10]. Despite the promising findings, there remains a notable lack of smoking cessation support provided by SCS professionals [9, 14]. To make significant progress in these organizations, it is important to understand the underlying factors contributing to this lack of support and factors that may facilitate improvement. However, as of now, no overview of the evidence is available on these factors. The aim of this study was to conduct a systematic review on the facilitators and barriers perceived by professionals in SCS settings to provide smoking cessation support. By identifying these factors, this review seeks to inform future initiatives designed to enhance smoking cessation support within SCS settings.

## Methods

### Search strategy

A systematic review was performed in February 2023 using the following databases: PubMed, PsycINFO, Scopus, CINAHL, and Cochrane Library. A search update was conducted to include studies up to April 2024. All papers identified in our searches were exported to Rayyan, to facilitate the screening process [20]. Keywords included terms for SCS settings (e.g. ‘community service’ or ‘social work’) and smoking cessation (e.g. ‘smoking’ and ‘tobacco’). The full search is shown in Supplementary Material S1. This systematic review was guided by the Preferred Reporting Items for Systematic Reviews and Meta-Analyses (PRISMA) (Supplementary Material S2) [21].

### Inclusion and exclusion criteria

The studies were selected with the following inclusion criteria: the study (1) used qualitative or mixed-method research designs presenting primary data analysis and using any recognized qualitative method of data collection; (2) included professionals working in SCS settings (i.e. individuals such as social workers and debt counsellors, providing support in areas such as work, participation, self-reliance, well-being, and youth, aiming to enhance the quality of life and enable full societal participation for disadvantaged groups) [18]; (3) focused on facilitators and barriers in providing smoking cessation support or implementing smoking cessation services; (4) was peer-reviewed.

The exclusion criteria were as follows: the study (1) only included professionals working in primary care or other healthcare settings; (2) only included students as participants; (3) did not distinguishing between smoking cessation and other health topics; (4) was published before 2000. The cut-off of the year 2000 was chosen due to the significant changes in tobacco control and smoking cessation support since the 20th century, which make older studies much less relevant to the goal of our research.

### Study selection

The screening process was guided by inclusion and exclusion criteria, during both the screening of titles/abstracts and the screening of full-text records. Initially, the first author (J.V.) independently screened all records based on titles and abstracts, while the second author (F.N.) independently screened 40% of these records on title and abstract. The discrepancy was less than 10% at this stage and was discussed until agreement was reached. Subsequently, the first author (J.V.) independently conducted full-text screening for the remaining records, while the last author (M.K.) screened 40% of these records. There was no discrepancy at this stage. The final step involved the screening process of the updated search, conducted by the first author (J.V.), who screened records based on titles and abstracts, followed by a full-text screening. If the first author (J.V.) had any doubts regarding the inclusion or exclusion of records, the last author (M.K.) was consulted.

### Quality assessment

The quality assessment of all included articles was performed by the first and last author (J.V. and M.K.). Discrepancies between the two authors were discussed until consensus was reached. To assess the methodological quality of the included studies, the mixed methods appraisal tool (MMAT) version 2018 was used, focusing on the category of qualitative studies [22], which consists of five items. Each item is rated ‘yes’, ‘no’ and ‘cannot tell’. The number of items rated ‘yes’ is counted to provide an overall score (0 is low; 5 is high). Studies with a low MMAT score (i.e. 2 or lower) were included, but only to compare findings with those from higher-quality studies and to support the findings of these studies. In Supplementary Material S3, we briefly specify how each criterion was interpreted and applied in the current review.

### Data extraction and synthesis

To extract data, the first author (J.V.) designed a specific form in which information of included articles was collected according to the authors’ name, year of publication, country origin of data, study methodology, sample, participants’ position, setting, and main findings. To analyse the data, a thematic synthesis of the included studies was performed [23, 24]. MAXQDA 2022 was used to analyse the results section of each paper. First, the first author (J.V.) carefully read the findings of the studies, including participants’ quotes and authors’ reporting of the findings. Then, all these excerpts were inductively coded. Next, authors J.V., A.R., and M.K. together discussed the codes, and grouped them into a structured model of themes and sub-themes. Discussions were conducted until reaching consensus on all (sub-)themes. After developing the (sub-)themes, the fourth author (A.K.) reviewed it, followed by discussions between J.V. and A.K., after which the (sub-)themes were refined and finalized.

**Figure 1:**
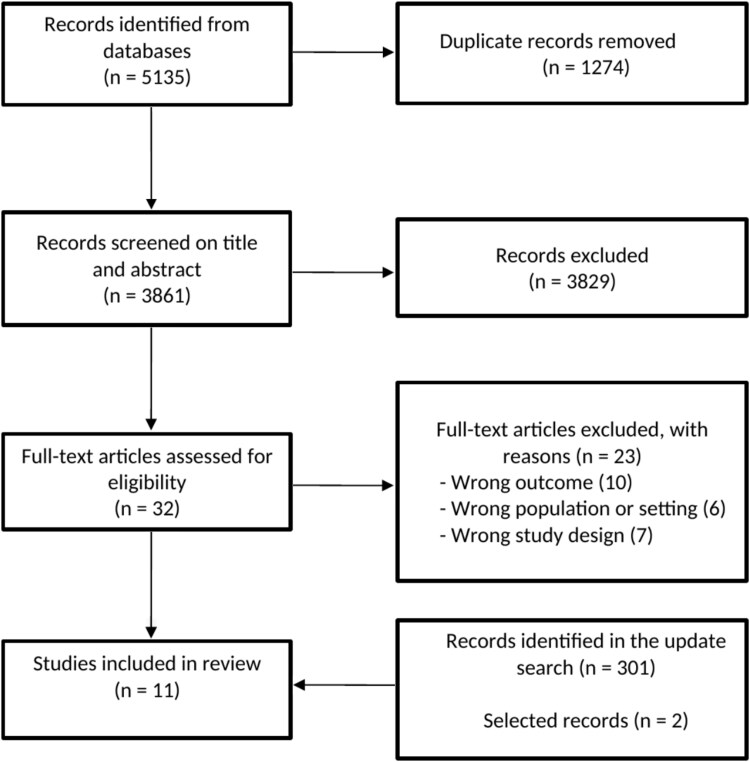
PRISMA diagram of the selection of included studies.

Themes were classified into four levels inspired by the Social Ecological Model: intrapersonal level (factors related to individual professionals), interpersonal level (factors that professionals attribute to clients or to the interaction between professional and client), organizational level (factors that professionals attribute to SCS organizations), and societal level (factors that professionals attribute to policies and processes outside the control of individuals and organizations) [25]. The Social Ecological Model acknowledges that an individual’s behaviour (e.g. providing smoking cessation support) is not solely determined by internal factors, such as personal beliefs and attitudes, but also by influences arising from social and ecological environments where an individual is situated.

## Results

### Study selection and characteristics

We found 5135 records in all databases. First, 1274 articles were detected as duplicates by Rayyan [20], checked by the first author and removed. Then, after screening 3861 records by title and abstract, 32 articles remained. Next, after studying the full text of the remaining articles, nine studies met the inclusion criteria. Reasons for exclusion of the articles were, e.g. focusing only on primary care professionals in primary care settings or focusing on clients’ barriers in smoking cessation. Lastly, in the updated search, we found 301 records. After screening these records, we identified two additional studies, bringing the total to eleven studies that met the inclusion criteria. Figure 1 shows the flowchart of the selection process.

The characteristics of each included paper are presented in Table 1. These articles, published between 2003 and 2024, were conducted in various countries: the United States (*n* = 2), Australia (*n* = 3), Belgium (*n* = 1), The Netherlands (*n* = 1), the United Kingdom (*n* = 2), Vietnam (*n* = 1), and a combined study in Bangladesh, Nepal, and Pakistan (*n* = 1). Five were mixed-methods studies and six were qualitative studies. Eight studies were exclusively conducted in community settings, such as community welfare organizations [18, 26–32], while three papers included both community and primary care settings, such as community and inpatient services [33–35].

Of the 11 papers included, seven specifically focused on the facilitators and barriers to provide smoking cessation support [18, 26, 28, 30, 31, 33, 35], while four focused on the facilitators and barriers to implement cessation programmes within SCS [27, 29, 32, 34]. As outcome measures, seven studies used the concept of facilitators or positive factors and barriers or negative factors [18, 28, 31–35], the four other studies examined the perspectives or attitudes of professionals, from which we derived information on facilitators and barriers [26, 27, 29, 30].

### Quality of included studies

Table 2 presents the results of the quality assessment. For both qualitative and mixed-methods studies, the qualitative methodological approach was appropriate to answer the research question. The majority of studies made use of qualitative data collection methods that were adequate to address the research question. However, in two studies, the content of the data collection materials (e.g. interview guide) remained unclear, and the method of analysis was not well explained [29, 34]. In eight studies, the interpretation of results was well-supported by the data. Nonetheless, in three studies there seemed to be lack of in-depth comparison and interpretation of the qualitative results with existing literature [29, 32, 34]. Coherence throughout the article was observed among most studies. However, in one study, the overall conclusion did not seem to align with the results presented earlier in the paper [32]. In total, two studies had a score as low as 2, meaning that they were only used to compare to and support the results found in the other studies [29, 34].

**Table 1:** Characteristics and main findings of the included studies.

**Authors and year**	**Country**	**Aim**	**Sample**	**Study design**	**Methodology of qualitative data collection**	**Participants’ position**	**Setting**	**Main findings**
Aquilino, Goody and Lowe, 2003 [26]	United States	To examine the perspectives of Women, Infants and Children (WIC) clinic providers on offering smoking cessation interventions for pregnant women	*N* = 25	Qualitative	Focus groups	Social workers, WIC nurses and dietitians	WIC clinics	Barriers: time, clinic priorities, staff approaches to clients, concerns about available educational material, client concerns (i.e. daily life demands, clients’ environment, offensive client), the absence of mechanisms to track clinic outcomes Facilitators: education material, information updates, additional training
Begh *et al.*, 2011 [27]	United Kingdom	To explore the perspectives of outreach workers on a community-based, outreach worker delivered, intervention that aimed to increase uptake of NHS smoking cessation services	*N* = 8	Mixed-methods (qualitative and quantitative)	Focus groups, interviews	Community outreach workers, local stop smoking service managers, specialist stop smoking advisor	Community setting	Barriers: feeling deskilled, people lack interest in quitting smoking, people have negative attitude of support, difficulties of allocating sufficient time, lack of suitable services, language barriers, people fear of family and friends Facilitators: building relationship, gaining trust and respect, same ethnicity and/or religion, sufficient time
Blumenthal, 2007 [33]	United States	To explore experiences and barriers to cessation services among community-based clinicians that serve minority or low-income populations in Georgia	*N* = 82	Qualitative	Focus groups	Social workers, physicians, physician assistants nurse practitioners, nurses, dietitians, administrators, pharmacists, and medical students	Community health centers, hospital setting, local public health department, private practices	Barriers: lack of time, inadequate cessation clinical skills, patient unreadiness to change, inadequate patient resources, inadequate provider resources Facilitators: additional training education material, NRT in organization, funding to set up programmes, smoking cessation treatment covered
Bryant *et al.*, 2010 [28]	Australia	To explore the perceptions of community welfare service managers, staff and clients about the acceptability of providing and receiving cessation support, (ii) organizational barriers to providing support and (iii) the types of support considered appropriate and feasible	*N* = 75	Mixed-methods (qualitative and quantitative)	Focus groups, interviews	Staff, managers and clients	Non-government community welfare organizations	Barriers: no priority, client in crisis, smoking is not core business, smoking is not on the radar, inadequate staff time, inadequate training, skills and knowledge, fear of worsen relationship, smoking is coping strategy for stress Facilitators: clients’ request for help, time available, trusting relationships, client’ willingness to quit, environment of trust, setting to identify smoking clients

*(continued)*

**Table 1:** Continued.

**Authors and year**	**Country**	**Aim**	**Sample**	**Study design**	**Methodology of qualitative data collection**	**Participants’ position**	**Setting**	**Main findings**
Hull *et al.*, 2012 [29]	Australia	To assess the effectiveness of a grants programme focusing on both the acceptability and feasibility of addressing smoking, as well as identifying changes in policy, organizational activities and staff attitudes	Unknown	Mixed-methods (qualitative and quantitative)	Document analysis of final project reports and case study report based on key informant interviews	SCSO staff, project coordinators	Non-government social and community service organizations	Barriers: frequent staff turnover, difficulties in filling vacant positions, limited resources available for recruiting highly skilled professionals, staff attitude: smokers rights
Leta *et al.*, 2023 [30]	Belgium	To gain insight smoking prevention within social work organizations from the perspectives of youngsters’ and youth workers’ perceptions	*N* = 46	Qualitative	Focus groups, interviews	Youth workers and youngsters	Social work settings providing sports-based and recreational activities (SR-settings)	Facilitators: accessible location, secure and encourage environment, knowledge of conversation skills, feeling of trust, extra activities/projects which buffer against smoking, youth workers as role models
Parker, McNeill and Ratschen, 2012 [34]	United Kingdom	To develop and implement a tailored tobacco dependence service in mental health settings and to assess its impact, as well as barriers and facilitators to implementation	Unknown	Mixed-methods (qualitative and quantitative)	Structured recording sheets, discussions	Staff of services, project team	Community and inpatient services	Barriers: lack of trust policy and guidelines, NRT not available, lack of cessation resources, lack of knowledge and skills, patient not ready to quit, smoking is coping strategy Facilitators: comprehensive staff training, dissemination of the audit and interim project results, development of cessation resources, close liaison with management and consultants, flexible, responsive approach to patients’ needs
Parnell *et al.*, 2020 [31]	Australia	To explore factors influencing community service organization (CSO) staff members’ willingness to provide tobacco cessation support to clients experiencing disadvantage	*N* = 29	Qualitative	Interviews	Community service organizations professionals	Community service organization setting: alcohol and other drugs, homelessness, and mental health sectors	Barriers: not a priority relative to other issue, being a current smoker, lack of knowledge and skills, lack of a formal tobacco cessation programme within the organization, lack of resources to assist staff, lack of consistent funding, client does not raise the topic, client is not ready to quit, smoking is a coping strategy Facilitators: client raises the topic, established relationship, gaining trust, confidence to provide support, training, organizational processes requiring staff to routinely ask clients about tobacco use, being a past smoker with experience

*(continued)*

**Table 1:** Continued.

**Authors and year**	**Country**	**Aim**	**Sample**	**Study design**	**Methodology of qualitative data collection**	**Participants’ position**	**Setting**	**Main findings**
Shelley *et al.*, 2014 [32]	Vietnam	To examine the attitudes and beliefs among Village Health Workers (VHWs) towards expanding their role to include delivering smoking cessation interventions and potential barriers and facilitator associated with implementing a VHW-delivered cessation intervention	*N* = 29	Qualitative	Focus groups	Community health workers (referred to as village health workers in Vietnam)	Community health centers, community settings	Barriers: lack of knowledge/ skills, lack of training, no priority, available time, lack of policies, lack of available national programmes, lack of media campaigns on cessation Facilitators: need for training, need for financial support, unique relationship, feeling of trust, knowledge of the smoking population, confidence in expanding their role, consistent with scope of work, social support clients’ environment, tobacco cessation information campaigns, collaboration with organizations
Visser *et al.*, 2024 [18]	The Nether-lands	To identify potential activities, barriers and facilitators in providing smoking cessation support in social and community organizations	*N* = 21	Qualitative	Interviews	Professionals from social and community service organizations	Social and community setting	Barriers: clients’ readiness and social environment, topic sensitivity, professional is non-smoker, lack of knowledge, no necessity, no responsibility, lack of priority, lack of time, lack of availability of appropriate services or high threshold referral process Professionals consider most themes facilitators if present and barriers if absent (except for clients’ environment and professionals non-smoking status), self-efficacy
Warsi *et al.*, 2019 [35]	Bangladesh, Nepal, and Pakistan	To understand factors affecting (tuberculosis) TB health workers’ delivery of tobacco cessation behavioural support, and subsequently developed a training package for LMICs	*N* = 25	Mixed-methods (qualitative and quantitative)	Focus groups, interviews	Low- and middle-income health workers: health workers, district- and central-level national tobacco control staff, facility in-charges	Bangladesh: an urban NGO-run clinic and a rural government centre Nepal: an urban, NGO-run, national referral center and a smaller, urban, government-run clinic Pakistan: a large, urban, government-run tertiary hospital and a rural, government-run hospital	Barriers: lack of knowledge, lack of confidence, inadequate communication skills, lack of cessation resources, lack of staff support (i.e. supervision and monitoring) lack of training, low prioritization/no agenda item, limited counselling time, high workload Facilitators: knowledge, provide training, staff support (i.e. supervision and monitoring), provide professional incentives (e.g. additional payments), need for manpower, release staff from other duties, more confidence, adequate workspace, request for help

**Table 2:** Results of the quality assessment using the MMAT version 2018.

	**Is the qualitative approach appropriate to answer the research question?**	**Are the qualitative data collection methods adequate to address the research question?**	**Are the findings adequately derived from the data?**	**Is the interpretation of results sufficiently substantiated by data?**	**Is there coherence between qualitative data sources, collection, analysis and interpretation?**	**Total score** ^**a**^
Aquilino *et al.* [26]	Yes	Yes	Yes	Yes	Yes	5
Begh *et al.* [27]	Yes	Yes	Yes	Yes	Yes	5
Blumenthal *et al.* [33]	Yes	Yes	Yes	Yes	Yes	5
Bryant *et al.* [28]	Yes	Yes	Yes	Yes	Yes	5
Hull *et al.* [29]	Yes	Can’t tell	Can’t tell	No	Yes	2
Leta *et al.* [30]	Yes	Yes	Yes	Yes	Yes	5
Parker *et al.* [34]	Yes	Can’t tell	Can’t tell	No	Yes	2
Parnell *et al.* [31]	Yes	Yes	Yes	Yes	Yes	5
Shelley *et al.* [32]	Yes	Yes	Yes	No	No	3
Visser *et al.* [18]	Yes	Yes	Yes	Yes	Yes	5
Warsi *et al.* [35]	Yes	Yes	Yes	Yes	Yes	5

a Total score is an indicator of study quality, with higher scores reflecting higher quality. Studies with a score of 2 or lower were considered low quality.

### Synthesis findings

Thematic synthesis identified eleven main themes, which were divided into levels inspired by the Social Ecological Model. These themes represent both facilitators and barriers for smoking cessation support in SCS settings (see Table 3), meaning that while some factors are perceived as facilitators or have the potential to facilitate the process, they may not always be present, which then forms a barrier. In the findings, both facilitators and barriers for each theme are grouped and discussed together, with the facilitators described first, followed by a paragraph outlining the barriers.

**Table 3:** Facilitators and barriers.

Level	Theme	Facilitators	Barriers
		Subtheme	Subtheme
Intrapersonal	Knowledge/skills	Knowledge/skills how to address smoking (cessation) Knowledge of the available smoking cessation services Knowledge about the background of a smoking client	Lack of awareness of the topic of smoking Lack of knowledge/skills to address smoking Lack of knowledge of the available smoking cessation services Lack of knowledge of smoking in general
	Self-efficacy	Belief about capacity to provide effective support Professional is (past) smoker	No belief about capacity to provide effective support Professional is non-smoker
	Belief (regarding topic of smoking within their field)	Belief that cessation support fits the scope of work Belief that their organization is a well-suited place for clients	Belief that cessation support does not fit the scope of work Belief that other problems are more urgent Belief that smoking is needed to deal with other problems Belief that smoking is clients’ own choice
Interpersonal	Trustworthy connection with client	Building a relationship with a client Feeling of trust between client and professionals Cultural similarities	Afraid to damage the relationship with a client Client is guarded Cultural differences
	Readiness of clients	Request for help from a client Client is willing to quit	No request for help from a client Client is not willing to quit Client has other priority Client has little confidence in quitting smoking Client has no need for support
	Clients’ supportive social environment	Social support in clients’ social environment	Fear for negative reactions from clients’ social environment Smoking behaviour in clients’ social environment
Organizational	Expertise improvement in smoking cessation	Training on how to provide smoking cessation support Updates on general information about smoking	Lack of training to provide smoking cessation support
	Availability of resources	Recording instruments (to document smoking) Education material for clients Smoking cessation programmes within the organization NRT available within the organization Availability of trained professionals Additional activities supporting smoking cessation	Lack of recording instruments (to document smoking) Lack of education material for clients Lack of available smoking cessation programmes within the organization Lack of available NRT within the organization Lack of trained professionals
	Organization support	Organizational attention to smoking cessation Time allocated to provide support Financial incentives for professionals Adequate workspace for professionals Collaboration between organizations	Lack of organizational attention Lack of time to provide support Lack of financial incentives for professionals Lack of adequate workspace Lack of implementing guidelines
Societal	Availability of cessation programmes	Availability of appropriate smoking cessation programmes	Lack of appropriate smoking cessation programmes
	Supportive healthcare financing	Funding for smoking cessation programmes	Lack of funding for smoking cessation programmes Treatment not covered by health insurance
	Public awareness	Media campaign on smoking Smoking is a topic on the political agenda	Lack of media attention on smoking cessation Lack of smoking as a topic on the political agenda

#### Intrapersonal level

*‘Knowledge/skills’* was identified as one of the themes on the intrapersonal level [18, 26, 28, 30–35]. Professionals stated that the understanding of the target population who use social services (often with a lower SEP) facilitates them to engage clients in smoking cessation [32, 35]. In addition, this also applies to their knowledge and skills to discuss topics such as smoking and their knowledge of the available services to which they can refer clients to [18, 30, 31, 35]. For instance, some professionals mentioned that they know how to properly interact with clients, ensuring that clients would not feel judged. This enables them to discuss sensitive topics such as smoking. *‘I think it is important to start the conversation that it is just not healthy and to be able to give information somewhere in a good way. But also not a whole explanation of this*  *is bad’* [30].

However, while some professionals felt they already had the necessary skills to address smoking cessation and knew where to refer clients, this was not the case for all professionals [18, 26, 28, 31–35]. For example, various professionals reported a general lack of knowledge about the topic of smoking [26, 32, 34, 35]. In addition, some professionals mentioned that the topic is not top of mind for them, resulting in it receiving no attention [18, 28]. *‘If you had a visitor, or maybe you have in the past, that wanted information or help to quit smoking—where would you send them?’* [31].

Another theme identified was *‘self-efficacy’* of the professional [18, 28, 31, 32, 35]. Professionals’ belief in their capacity to perform well was indicated as an important facilitating factor [18, 31, 32, 35]. In addition, they also emphasized the importance of personal smoking experience, which would enhance the belief in their capacity to empathize deeply and understand the challenges faced by smoking clients [31]. Both factors have lowered the threshold for professionals when addressing smoking in the past. *‘A lot of the conversations go, “You should quit smoking.” And then they sort of go along the line of, “How do you know anything about it?” “Well, actually I do … trust me, you”ll be better off’* [31].

Nonetheless, while several professionals felt confident in their ability to effectively address smoking, others expressed concerns about their capacity to provide adequate support for smoking cessation [28, 31]. Furthermore, lack of personal experience with smoking contributed to these concerns, as they fear they may misunderstand smoking clients [18, 28]. *‘I don’t know how well skilled I am, confident I would feel, giving advice about stopping smoking’* [28].

*‘Professionals’ beliefs’* about smoking as an issue within their field emerged as a key theme influencing their efforts in smoking cessation [18, 26, 28–32, 34, 35]. Some believe that smoking cessation improves the health and well-being of clients, therefore seeing support in smoking cessation as their responsibility, as it aligns closely with their overall job focus [18, 28, 32]. Furthermore, some professionals believe that it is not only well-suited to their job focus but also to their workplace [30]. *‘An additional role comes into play when problems in other life domains cause financial instability. If unhealthy behaviour leads to an inability to manage debts, then you have an extra responsibility’* [18].

However, while some professionals viewed these beliefs as facilitating factors, not all shared the same perspective. In contrast, for others, their beliefs were seen as barriers to providing effective support. For example, various professionals believe that supporting smoking cessation is not necessarily part of their job description, leading them to feel no responsibility for it [18, 28, 35]. Moreover, due to the many challenges clients face, professionals often believe that other issues are more urgent [18, 26, 28, 31]. Sometimes, they even believe that smoking is their personal choice or necessary to cope with these problems [28, 29, 31, 34]. *‘There would be time when we would actually discourage families from giving up smoking at that particular point in time, because of the high stress they’re under’* [28].

#### Interpersonal level

*‘Trustworthy connection with clients’* was identified as one of the themes at the interpersonal level [18, 26–28, 30–35]. The established relationship between professionals and clients, which is characterized by a feeling of trust, was identified as reason for being more willing to talk about a clients’ smoking behaviour [18, 27, 28, 30–32]. *‘It’s about building the relationship, building the rapport and as soon as they see that okay this person is here for my benefit then they will start opening up’* [27].

While the established relationship could be beneficial according to various professionals, some also viewed it as a barrier. They fear that advising clients to quit could damage the relationship or even result in losing a client, as it might be perceived as judgmental [18, 28]. The experience of professionals with clients responding defensively when bringing up the topic was hindering the initiation of conversations about smoking [26–28, 33–35]. *‘It’s one of those topics that’s hard to talk about [smoking] … they think you’re lecturing them on something bad and … [they] immediately get defensive’* [26].

Another theme identified was *‘readiness of clients’* [18, 26–28, 31, 33–35]. Professionals highlighted that if a client initiates a discussion about smoking, seeks help to quit, or expresses willingness to include quitting smoking in their goals, professionals are receptive to continue the conversation about smoking and provide support [18, 27, 28, 31, 33, 35]. *‘They’ve got to make the first step to say, “I want to quit”’* [31].

However, although clients’ readiness can facilitate initiating conversations about smoking, professionals often noted that clients rarely ask for help or perceive no need for support in quitting smoking [18, 27, 28, 31]. In addition, clients often have other issues they want to address during counselling or may not believe they can quit smoking while dealing with these issues [18, 26, 28, 31, 33]. Professionals also indicated that it is a challenge that some clients do not perceive smoking as a problem or do not want to quit [18, 27, 31, 33, 34]. *‘The biggest barrier I’ve gotten from patients is they enjoy smoking. So they don’t want to quit. So we work on that’* [33].

*‘Clients’ supportive social environment’* was identified as an important theme [18, 26, 27, 32], with the social environment mentioned as a valuable, additional source of support during the quitting process [32]. *‘We can only talk to smokers one or two times but their family members can talk to them more than that’* [32].

On the other hand, other professionals mentioned that a client’s social environment could be a barrier to, for example, advising them to quit smoking. Professionals highlighted that the smoking behaviours of those around clients, along with their potential negative reactions, could hinder the quitting process and make professionals feel powerless [18, 26, 27]. *‘People often find it challenging to admit to others that they may want to quit. In certain groups, individuals are sometimes ridiculed when they express a desire to stop smoking’* [18].

#### Organizational level

*‘Expertise improvement in smoking cessation’* emerged as an important theme [26, 28, 31–35], with professionals mentioning that training on how to provide smoking cessation support, including guidance, would be an important facilitator [26, 31–35]. They also emphasized the importance of receiving updates on general information about smoking, such as periodic updates on the latest research regarding the health effects of smoking and intervention options [26]. *‘We have a lot of things to do, but training is important … I don’t think many people [staff] are prepared to talk about smoking … I could use more information. There’s new stuff every day that relates to smoking, so I know there’s new and up-to-date stuff that we probably don’t know about’* [26].

However, while professionals view training and general information about smoking as important facilitators, they stated that there is currently a lack of adequate training, contributing to an environment where smoking cessation support is not prioritized or discussed professionally [28, 32, 35]. *‘We have not been trained on counselling … If we [health workers] had special counselling training, only then would we know [how to counsel properly]’* [35].

Another theme identified at the organizational level was the *‘availability of resources’* [26, 29–31, 33–35]. Professionals mentioned that recording instruments within their organization, such as tools to track the number of smokers or to record clients’ smoking behaviour, would encourage them to provide support [31, 34, 35]. The availability of educational materials and nicotine replacement therapy (NRT) within organizations was also mentioned [26, 29, 33–35]. In addition, alongside existing programmes that could facilitate clients’ smoking cessation process (e.g. programmes focusing on social skills or emotional regulation), having smoking cessation programmes available within the organization, including trained staff, would be beneficial [30, 31, 35]. *‘We need record reporting registers*  *[for tobacco use] or some cards. If we have such cards, we can report accordingly. If there is recording and reporting, work will be done properly …’* [35].

While professionals view the availability of resources, such as educational materials and recording instruments, as important facilitators, some of them indicated a current lack of standard recording instruments and educational materials for clients, as well as the absence of smoking cessation programmes and trained staff within the organization [26, 29, 31, 33, 35]. In addition, professionals mentioned that due to the lack of NRT within their organization they must refer clients to specialists to get it [33, 34]. *‘I don’t have any handouts that I consistently give. So if they’re young and I have some information from the American Lung Association, I may give that out, but nothing on a consistent basis, which is what we*  *need’* [33].

*‘Organizational support’* was identified as one of the themes at organizational level [18, 26, 28, 32–35]. Various professionals mentioned that if smoking would be part of the conversation within the organization, they would be more likely to be aware of the issue [18]. Other important facilitating factors mentioned regarding organizational support included organizations allocating time to address smoking behaviour, possibly offering additional salary, and providing good workspaces [18, 32, 35]. *‘… for proper and smooth work, financial assistance and certificates should be awarded, that is moral and financial support … because then work will be done with interest … in the proper way …’* [35].

Nonetheless, several professionals mentioned that within the organization, smoking cessation currently receives less attention and is not always seen as important as other issues, which is a barrier to providing support for smoking cessation [32, 35]. Moreover, there is an absence of clear guidelines or agreements regarding the role of professionals in smoking cessation efforts [18, 34]. In addition, there is a lack of time, adequate workspace or financial incentives allocated to these efforts [18, 26, 28, 32, 33, 35]. *‘Those 3 or 4 extra conversations that are sometimes needed to motivate someone to quit an addiction, well, I don’t have the time for that because I don’t get paid*  *for it’* [18].

#### Societal level

The *‘availability of appropriate smoking cessation programmes’* (outside the organization) was identified as one of the themes at societal level [18, 27, 32]. Professionals emphasized that the availability of appropriate smoking cessation programmes would be reassuring, as it enables them to provide helpful assistance when someone seeks help with cessation [18, 32]. *‘It is helpful to be able to refer someone*  *when you notice someone is struggling and having difficulty quitting’* [18].

Nevertheless, although appropriate smoking cessation programmes could be facilitating, there is often a lack of appropriate smoking cessation programmes provided by public health authorities. According to professionals, there is a lack of appropriate smoking cessation programmes to help smokers quit as they are often too hurried, formal or impersonal [18, 27, 32]. ‘*It’s more difficult when you [speaking from a client’s perspective] have to initiate contact [with the smoking cessation service]; you might even think: Never mind, it’s not necessary’* [18].

*‘Supportive healthcare financing’* emerged as an important theme [18, 29, 31, 33]. Professionals emphasized the importance of the government allocating funds to SCS organizations to set up smoking cessation programmes [33]. This financial support would encourage these organizations to expand their efforts in providing smoking cessation support. *‘If the state would allocate money to the community health centres to set up smoking cessation programs, then it would be beneficial to us. We could, if we had the funds available, hire 1 or 2 people that would do nothing but smoking cessation programs …’* [33].

Nonetheless, professionals highlighted that there is most often a lack of funding for organizations to set up smoking cessation programmes within the organization [18, 29, 31]. For example, limited funds restrict the recruitment of highly skilled professionals, and many smoking cessation programmes have had to end due to budget cuts. If programmes within the organization are not feasible due to a lack of funding, referring individuals to external programmes can also be challenging. Not only must such programmes exist, as previously described, but they should also be affordable. In the United States, professionals highlighted the issue of clients who are ready and motivated to quit smoking, only to discover that their insurance does not cover the necessary expenses [33]. This problem is particularly prevalent among low-income clients, who often cannot afford support. *‘The quit smoking program that we had before, it’s something I think that needs to be consistent … When you only get funding for it to run a couple of times, it’s not going to work, I don’t*  *think’* [31].

*‘Public awareness’* was identified as one of the themes at the societal level [18, 32, 35]. Professionals mentioned that including smoking as a topic on the political agenda may increase their awareness of the issue [18, 35]. They also highlighted that the ongoing media campaigns, including warning labels on cigarette packages and smoke-free air policies, play an important role in educating clients about the dangers of tobacco use [32]. *‘If smoking is a priority above us [government] or more often part of the conversation, it will definitely reach the professionals’* [18].

However, although including smoking on the political agenda may raise awareness, professionals noted its current absence as a prominent item on the national government’s agenda [32, 35]. In addition, while media campaigns emphasize the dangers of tobacco use, there remains a significant lack of focus on smoking cessation in the mass media messages [32]. *‘The information broadcasted on mass media focuses on the dangers of tobacco only, not the methods of tobacco cessation’* [32].

## Discussion

### Key findings

The aim of the study was to explore facilitators and barriers perceived by professionals working in SCS settings to provide smoking cessation support. We found that various factors together might encourage and facilitate professionals in providing smoking cessation support. These factors were related to the professional (i.e. knowledge and skills, high self-efficacy, and positive belief towards smoking cessation), client (i.e. trustworthy connection with clients, readiness of clients, clients’ supportive social environment), organization (i.e. expertise improvement in smoking cessation, availability of resources, and organizational support), and society (i.e. availability of cessation programmes, supportive healthcare financing, and public awareness). However, this review also showed that, in practice, these factors oftentimes do not act as facilitators, due to their absence, thus hindering professionals from providing such support.

### Interpretation of the findings

We assigned the different facilitators and barriers to different socio-ecological levels. While relevant factors thus operate at different levels, these levels are interconnected. For instance, we found that many clients do not explicitly request help with quitting smoking and that professionals assume that these clients are not willing to quit (interpersonal level)*.* However, various studies have reported that two-thirds of current smokers have a desire to quit [36], suggesting that professionals may not be sufficiently aware of clients’ willingness to quit smoking (intrapersonal level). Offering targeted training within SCS settings may address this awareness gap and encourage professionals to proactively engage in conversations about smoking with their clients (organizational level) [37].

The evidence from this review suggests that addressing barriers at the organizational level may be crucial, and that the improvement of expertise in smoking cessation may be particularly important to improve smoking cessation support. Previous research has demonstrated that integrating training within an organization can enhance professionals’ knowledge and skills, as well as positively influence their beliefs regarding smoking cessation and their roles in supporting it [10, 37]. Nevertheless, training alone may be insufficient to achieve lasting change [37, 38]. For an innovation to be successfully embedded, it is essential to have a supportive organizational environment. The Addressing Tobacco through Organizational Change model provides a structured guidance to help organizations change so that they can support clients in smoking [38]. This includes changes that directly impact the professionals who may provide support, such as implementing training, guidelines, and the provision of educational materials [38]. Furthermore, it includes changes to the broader organizational environment, such as policies on limited smoking hours, prohibiting staff from smoking with patients, and implementing smoke-free grounds [38]. Previous research has demonstrated that multi-level changes in SCS organizations improve organizational support for smoking cessation training and resources, smoking-free policies, and the provision of free NRT [29]. Nonetheless, integrating smoking cessation treatment into an organization may be challenging [38, 39].

The assembled evidence implies that governments could support smoking cessation efforts by providing funding to SCSs dedicated to these initiatives. This is important as this study revealed that professionals often do not feel supported by their superiors, even though they want to provide smoking cessation support. This lack of prioritization serves as a barrier to their effective engagement in these efforts. Previous research has shown that the provision of grant funding can enhance the acceptability and feasibility of addressing smoking within SCS organizations, as well as changes in tobacco policy and professionals attitudes [29]. In addition, government could facilitate the availability of effective smoking cessation programmes, which may encourage SCS professionals to refer their clients to these programmes. Importantly, such smoking cessation programmes should be tailored to the needs of people from lower SEP groups, emphasizing elements such as cost-free access and the provision of free NRT [8, 40]. While smoking cessation treatments may be covered by public health systems, there are large variations in which treatments are reimbursed, with which frequency this is possible, whether all costs are covered and whether upfront payment is required. For example, in 2022, 66% of WHO European Region countries covered NRT or cessation services [41]. Access to specific services, medications, or timely interventions tailored to the needs of low SEP individuals may still be limited or not covered, even in health systems that do facilitate free smoking cessation services. Importantly, the short-term costs for the services may be compensated in the long term thanks to increased quit success rates and ultimately reduced burden of smoking-related disease and health care demand [40, 42].

One might question whether smoking cessation support should be integrated into SCSs or whether it should be the exclusive responsibility of primary health care. A recent systematic review on barriers and facilitators among health professionals in primary care for the prevention of cardiometabolic diseases, including supporting smoking cessation, has revealed barriers and facilitators that are highly comparable to those identified in this review [43]. Interestingly, one difference is that our review findings highlighted that SCS professionals could align smoking cessation services with existing SCS services that focus on managing emotions or developing social skills. This might significantly increase clients’ smoking cessation success rates, given that people with a lower SEP often smoke as a coping mechanism to stressful living conditions, such as unemployment, financial stress and poverty, and these programmes can specifically address the underlying reasons for clients’ smoking behaviours [9, 44]. Further research is needed to compare the effectiveness of smoking cessation support provided in SCS settings with that offered in primary care settings.

### Strengths and limitations

This is the first review that offers a comprehensive overview of challenges to provide smoking cessation support in SCS settings to people with a low SEP. However, various limitations should be considered when interpreting our results. First, two out of eleven studies did not distinguish between SCS professionals and primary care professionals. These studies may not accurately reflect the perspectives of only SCS professionals. Second, our results are heterogenous: studies from different countries do not consistently identify the same facilitators and barriers, as the operation of SCS organizations varies across countries. The implementation of smoking cessation as a topic in these organizations is highly dependent on both local and national healthcare systems, including the organization of funding and specific goals. Therefore, although we have been able to give a general overview of relevant factors, the way to operate in practice may vary across countries. Third, two studies were assessed as being of low quality, raising questions about the validity of their results. Therefore, we have chosen to use these findings solely to support the overall results, resulting in a limited overall impact. Finally, a limitation is that the search string may be limited, as ‘social and community services’ is a broad term that includes different types of organizations and roles, across various countries, which may have resulted in some relevant studies not being captured.

## Conclusion

Professionals working in SCS settings may be in a position in which they can effectively reach many people with a lower SEP who smoke. However, oftentimes many barriers prevent them from offering smoking cessation support. To fully harness their potential, organizational changes are necessary, such as implementing training programmes to enhance professionals’ skills in addressing smoking, establishing guidelines for professionals’ role in providing smoking cessation support, providing educational materials on tobacco dependence and treatment, and instituting policies for restricted smoking hours or a complete ban on smoking among professionals.

## Supplementary Material

Supplementary_material_1_cyaf030

Supplementary_material_2_cyaf030

Supplementary_material_3_cyaf030
