# Supplementary material for: Facilitators and barriers to smoking cessation support among professionals in social and community service settings: a systematic review and thematic synthesis
Source: Health Educ Res. 2025 Aug 12;40(5):cyaf030. doi: 10.1093/her/cyaf030 (PMC12343063; doi:10.1093/her/cyaf030)
Supplement: Supplementary_material_1_cyaf030 [file supplementary_material_1_cyaf030.doc]

*Supplementary Material 1 – detailed search string.*

**PsychINFO search**

social service.mp.

community service.mp.

community organisation.mp.

community organization.mp.

community service organisation.mp.

community service organization.mp.

social welfare.mp.

welfare work.mp.

welfare worker.mp.

social welfare organisation.mp.

social welfare organization.mp.

exp Social Workers/

social worker.mp.

community worker.mp.

social work.mp.

exp Smoking Cessation/

Smoking.mp.

Tobacco.mp.

Smoker.mp.

1 or 2 or 3 or 4 or 5 or 6 or 7 or 8 or 9 or 10 or 11 or 12 or 13 or 14 or 15

16 or 17 or 18 or 19

20 and 21

**Scopus**

TITLE-ABS-KEY ( ( ( "social service" ) OR ( "community service" ) OR ( "community organisation" ) OR ( "community organization" ) OR ( "community service organisation" ) OR ( "community service organization" ) OR ( "social welfare" ) OR ( "welfare work" ) OR ( "welfare worker" ) OR ( "social welfare organisation" ) OR ( "social welfare organization" ) OR ( "Social Workers" ) OR ( "social worker" ) OR ( "community worker" ) OR ( "social work" ) ) AND ( ( "Smoking Cessation" ) OR ( Smoking ) OR ( Tobacco ) OR ( Smoker ) OR ( "Tobacco Use" ) ) )

**CINAHL**

(AB social service OR AB community service OR AB community organisation OR AB community organization OR AB community service organisation OR AB community service organization OR AB social welfare OR AB welfare work OR AB welfare worker OR AB social welfare organisation OR AB social welfare organization OR AB Social Workers AB social worker OR AB community worker OR AB social work) AND (AB Smoking Cessation OR AB Smoking OR AB Tobacco OR AB Smoker OR AB Tobacco Use)

**PubMed search** ("social service*"[Title/Abstract] OR "community service*"[Title/Abstract] OR "community organisation*"[Title/Abstract] OR "community organization*"[Title/Abstract] OR "community service organisation*"[Title/Abstract] OR "community service organization*"[Title/Abstract] OR "social welfare*"[MeSh Terms] OR "social welfare*"[Title/Abstract] OR "welfare work"[Title/Abstract] OR "welfare worker*"[Title/Abstract] OR "social welfare organisation*"[Title/Abstract] OR "social welfare organization*"[Title/Abstract] OR "Social Workers"[MeSH Terms] OR "social worker*"[Title/Abstract] OR "community worker*"[Title/Abstract] OR "social work*"[Title/Abstract]) AND ("Smoking Cessation"[MeSH Terms] OR "Smoking Cessation"[Title/Abstract] OR "Smoking"[MeSH Terms] OR "Smoking"[Title/Abstract] OR "Tobacco"[MeSH Terms] OR "Smoker*"[Title/Abstract] OR "Tobacco"[Title/Abstract] OR "Tobacco Use"[MeSH Terms])

**Cochrane Library**

(social service):ti,ab,kw OR (community service):ti,ab,kw OR (community organisation):ti,ab,kw OR (community organization):ti,ab,kw OR (community service organisation):ti,ab,kw OR (community service organization):ti,ab,kw OR (social welfare):ti,ab,kw OR (welfare work):ti,ab,kw OR (welfare worker):ti,ab,kw OR (social welfare organisation):ti,ab,kw OR (social welfare organization):ti,ab,kw OR (Social Workers):ti,ab,kw OR (social worker):ti,ab,kw OR (community worker):ti,ab,kw OR (social work):ti,ab,kw AND (Smoking Cessation):ti,ab,kw OR (Smoking):ti,ab,kw OR (Tobacco):ti,ab,kw OR (Smoker):ti,ab,kw OR (Tobacco Use):ti,ab,kw
