# Supplementary material for: Facilitators and barriers to smoking cessation support among professionals in social and community service settings: a systematic review and thematic synthesis
Source: Health Educ Res. 2025 Aug 12;40(5):cyaf030. doi: 10.1093/her/cyaf030 (PMC12343063; doi:10.1093/her/cyaf030)
Supplement: Supplementary_material_3_cyaf030 [file supplementary_material_3_cyaf030.doc]

*Supplementary Material 3 – Description of MMAT for qualitative studies.*

| **Criteria for qualitative studies** | **Description** |
| --- | --- |
| 1. Is the qualitative approach appropriate to answer the research question? | We checked whether the qualitative approach used in the study is appropriate to answer the research question. To do this, we checked whether the research question is in line with and addresses the problem described in the study. |
| 1. Are the qualitative data collection methods adequate to address the research question? | We checked whether the method of data collection (e.g., in depth interviews and/or group interviews, and/or observations) and the form of the data (e.g., tape recording, video material, diary, photo, and/or field notes) are adequate and most appropriate to address the research question. This involved evaluating whether the selected methods facilitated gathering enough detailed and varied information. Also, clear justifications are needed when data collection methods are modified during the study. |
| 1. Are the findings adequately derived from the data? | We checked whether the data analysis method used in the study is appropriately addressed in the study. Also, we checked if the data analysis method used in the study is consistent with the qualitative approach. Further, we checked whether there is an appropriate explanation given for how findings (such as themes, concepts, categories, etc.) were derived from the data. |
| 1. Is the interpretation of results sufficiently substantiated by data? | We checked whether the interpretation of the results is supported by the data being collected. Also, we checked whether the results are well-supported by relevant literature to get insight in the validity of the results. Further, we checked whether important findings are not being discussed or whether aspects were discussed that had not been previously mentioned. |
| 1. Is there coherence between qualitative data sources, collection, analysis and interpretation? | We checked whether the qualitative data sources, collection, analysis and interpretation are consistent with each other. We checked this by looking at the clarity of the entire research process and assessing whether the aspects described did not emerge unexpectedly throughout the research. |
